# Study on factors influencing college students’ digital academic reading behavior

**DOI:** 10.3389/fpsyg.2022.1007247

**Published:** 2023-01-12

**Authors:** Liyan Chang, Yujie Wang, Jing Liu, Yao Feng, Xinyao Zhang

**Affiliations:** ^1^School of Information Management, Nanjing University, Nanjing, China; ^2^School of Management, Nanjing University of Posts and Telecommunications, Nanjing, China

**Keywords:** digital academic reading, structural equation model, influencing factors, behavioral intention, social influence, reading behavior, perceived risk

## Abstract

**Background:**

Affected by the COVID-19, many colleges have adopted online teaching. Meanwhile, the digital transformation of academic journals has shifted readers’ reading habits from traditional paper media to digital media. Digital academic reading has become the main reading method of college students during the COVID-19 pandemic.

**Purpose:**

The purpose of this study was to investigate the behavioral characteristics of college students’ digital academic reading and explore the internal factors and external environmental factors affecting the Intention and Use behavior of digital academic reading. At the same time this study provide recommendations to address these influencing factors in terms of the individual, the environment and library resources.

**Methods:**

Based on UTAUT2 model and digital academic reading theories, this paper constructs a digital academic reading information behavior (DARB) model of college students to examine college students’ digital academic reading behavior and intention. College students with digital academic reading behavior were recruited as research participants. A multi-stage sampling technique was used to collect representative samples from universities in Nanjing. 239 respondents participated in the questionnaire, with 189 providing valid data. Results: Effort expectancy (EE), social influence (SI), price value (PV), perceived risk (PR) and habit (BH) have a significant impact on behavioral intention (BI), and behavioral intention (BI) and habit (BH) have a significant impact on use behavior (B). However, performance expectancy (PE) and facilitating conditions (FC) have no significant influence on behavioral intention (BI).

**Originality/value:**

The findings of this study will help fill the gap in previous research on the relationship between the influencing factors of digital academic reading and college students’ reading intentions and behaviors, so as to provide a basis for improving the academic reading literacy program in colleges and optimizing the current digital academic reading environment.

## 1. Introduction

Academic reading is the manifestation of academic literacy and an important behavior to obtain academic resources and promote academic communication. In the process of university construction toward a higher level, improving the comprehensive quality of college students and cultivating their academic literacy are the basis for building high-quality universities and cultivate first-class talents. The arrival of the COVID-19 in 2019 and the implementation of epidemic prevention measures (such as home quarantine and the suspension of all educational institutions) have brought about unexpected changes in academic reading habits of college students. Many colleges have adopted online teaching during the COVID-19 pandemic, and college students have got accustomed to online learning (Maria and arios, 2022). Meanwhile, the advent of Internet plus era and the digital transformation of academic journals have changed the behavioral characteristics of college students’ reading (Sun et al., 2021). Digital academic resources have become an important source of academic reading for college students (Peng, 2017), and social networks have also become an important academic information sharing channel for them (Yang, 2019), digital academic reading has become the main reading method of college students during the COVID-19 pandemic. While university students are gaining a new digital reading experience, many problems have been revealed, such as the fragmentation and simplification of the use of digital reading, “addiction” and the prevalence of “information loss” (Gong et al., 2020). College students are the main group of digital academic reading and it is of significance to pay attention to the influencing factors of their digital academic reading behavior. Therefore, based on the UTAUT2 model, UTAUT model and TAM model, this paper expands, modifies and adds some factors specific to the academic reading environment, such as perceived risk, and constructs the digital academic reading information behavior (DACB) model of college students. The study analyzes the key factors affecting digital academic reading behavior through structural equation model, to provide a basis for schools to develop digital academic reading intervention strategies (e.g., increasing the price value of academic reading), to develop students’ academic reading literacy and to optimize the current digital academic reading environment.

## 2. Literature review

### 2.1. Definition of digital academic reading

“Academic reading” has been around for years and has been adopted by a variety of researchers. Ananda and Zhang (1987) put forward the concept of academic reading and first believed that it was a curriculum and an information behavior carried out by people in scientific research. Compared with traditional reading, digital reading is a new concept, which is similar to the concepts of electronic reading, online reading, virtual reading, ubiquitous reading and so on. Academic reading is a purposeful and critical reading, unlike idle reading completely based on personal preferences. Since then, some researchers have proposed different concepts of digital reading and academic reading, respectively. This paper integrates these two concepts to define digital academic reading.

According to the definition of the two concepts in existing studies, digital academic reading refers to a reading activity in which readers obtain information from professional books, academic papers, international conference papers and other academic documents and information content on academic forums with the help of digital carriers such as mobile phones, tablets, computers and other electronic products Table 1.

**Table 1:** Conceptual overview of digital reading and academic reading.

Concept	Proposer	Definition
Academic reading	Grabe and Stoller (2002)	Academic reading is a process in which readers understand the academic information obtained after browsing materials and papers, so as to form a knowledge framework.
	Hu and Chen (2014)	Academic reading refers to sorting and synthesizing the information obtained by reading various academic materials, so as to expand the vision of scientific research and improve one’s academic competence.
	Nie and Li (2018)	Academic reading is a kind of reading activity in which learners study the classic works and cutting-edge documents of their major in order to improve their professional qualities, expand the professional knowledge basic, improve professional research skills and build a scientific professional knowledge system.
	Liu et al. (2012)	Professional reading refers to the reading behavior of professionals in reading the professional or related materials.
	Manarin et al. (2015)	Academic reading is concerned with integrating knowledge into new knowledge and using discrete strategies to interpret academic texts.
Digital reading	Tang and Lin (2004)	Digital reading refers to the reading behavior of using digital intelligent devices to read hypertext, which is formed by multimedia integration.
	Bi (2010)	Digital reading is a process of acquiring or disseminating knowledge digitally, regardless of the tools, places or methods.
	Ke (2015)	Digital reading is a reading activity and cultural phenomenon based on the acquisition of digital text knowledge and digital media information.
	Baidu Baike	Digital reading includes two aspects: one is the digitization of reading objects, the other is the carrier of reading methods.
	Hahnel et al. (2016)	Digital reading is defined as proficiency in reading and understanding digital text organized in a non-linear format (called “hypertext”).

### 2.2. Development of digital academic reading

Academic Reading is a process of extracting and constructing meaning through interaction with written language (Kirby, 2003). And it helps students to seek specialized knowledge, access academic information, conduct academic exchanges, and finish their studies. Both college students and teachers believe that academic reading is very important (Howard et al., 2018; Gorzycki et al., 2020). However, nearly 60 percent of freshmen find that they are not academically prepared for higher education. Some countries and institutions have established a variety of assessment projects to determine students’ academic reading ability, such as NAAL (National Assessment of Adult Literacy), SAT (Scholastic Aptitude Test), ACT (American College Testing Programs) and so on. In multiple tests, approximately half of the students met the reading benchmark required by the college. Therefore, many researchers have conducted in-depth research on college students’ academic reading ability, attitudes, strategies, problems and challenges, behavioral regularities and influencing factors, and intervening measures to improve their reading skills.

With the development of the Internet, new media reading emerged around 2011 and digital reading has become way of learning and living for contemporary college students. Some scholars have long studied the difference between paper and screen reading (Dillon, 2007), and examined the media preference of undergraduate academic reading (Mizrachi, 2015). Xu and Li (2020) also studied the changes in students’ reading preferences and found that most of the college students still preferred to access information through paper carriers when reading relevant literature. A great number of studies have shown that digital reading environment presents new challenges and opportunities for learners’ reading ability. Guzmán-Simón et al. (2017) believed that undergraduate academic practice should include digital skills, ICT literacy and information literacy. What’s more, different reading forms and reading skills required for digital academic reading expanded the factors influencing academic reading of original paper to include factors such as the ICT environment and information skills. These results are supported by another study which states that the comprehending digital text requires different and additional skills and strategies (Reiber-Kuijpers et al., 2020).

In 2020, the outbreak of Covid-19 has changed people’s living conditions (Liu and Huang, 2020). Due to objective conditions, many colleges and universities have adopted online teaching, and Chinese college students have gradually developed digital academic reading habits. Global library access and circulation outlook indicates that physical access and circulation are on the decline, while digital access and circulation are on the rise. Although Covid-19 has created certain conditions for college students to engage in digital academic reading, the lack of information literacy and the limitation of digital academic resources have also brought difficulties (Hevia et al., 2021; Salim et al., 2022).

To summarize, the previous research mainly focused on the evaluation and teaching method of traditional academic reading (Cabrera-Pommiez et al., 2021; Yapp et al., 2021). Currently, the recent research on digital academic reading focuses on the definition of the digital academic reading, reading ability, academic literacies or digital learning platforms (Nhapulo et al., 2017; Diane and Alicia, 2022). Few scholars discussed the factors influencing college students’ digital academic reading behavior and willingness. This paper uses the UTAUT2 model to explore the impact of internal and external factors such as reading environment, technological factors, and reading attitudes on college students’ academic reading behavior.

## 3. Research model construction and hypotheses

### 3.1. Theoretical framework

**Figure 1:**
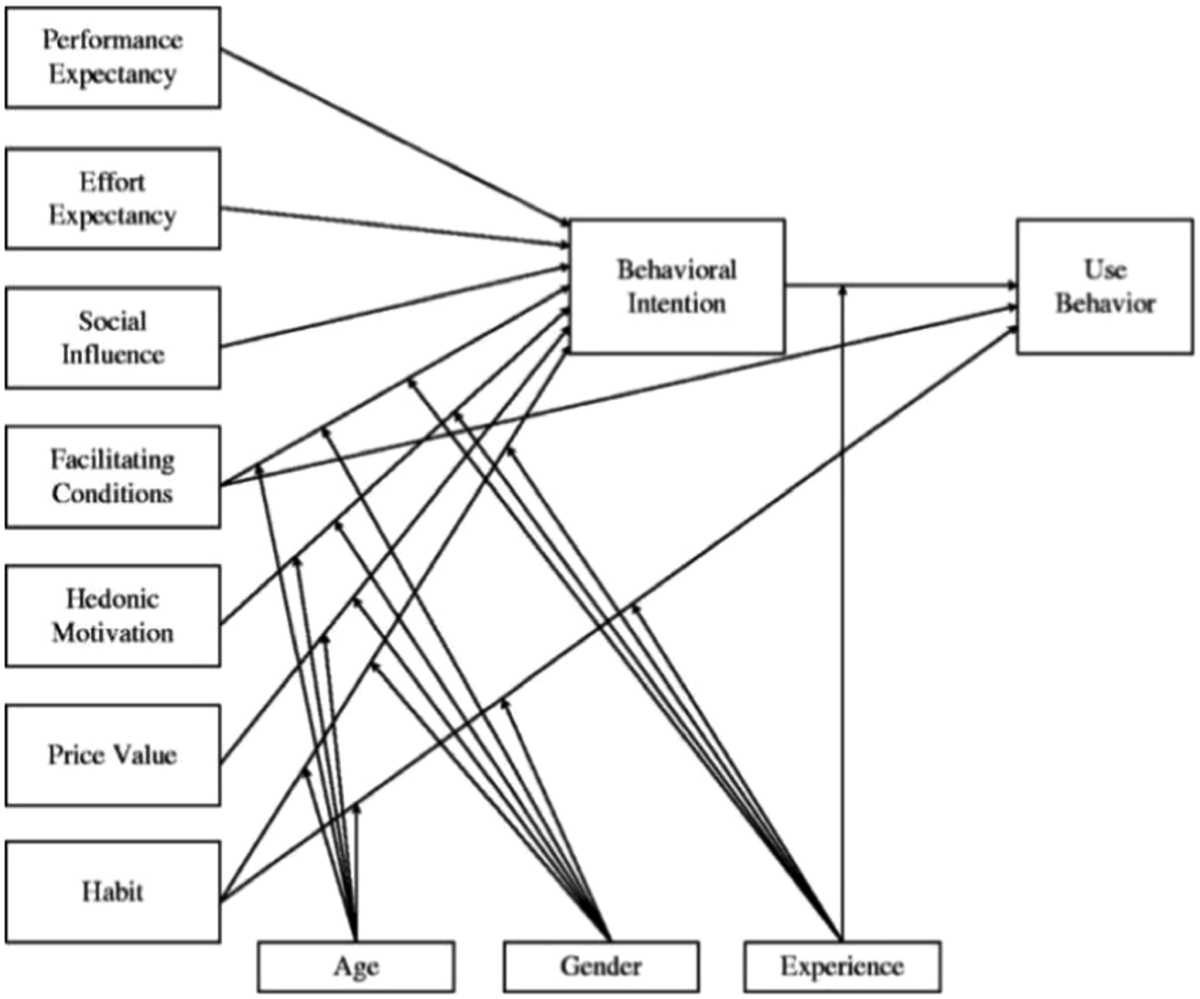
UTAUT2 model.

The research model for this study was the extended UTAUT2 with the addition of the Technology Acceptance Model (see Figure 1; Venkatesh et al., 2012). This model is more focused on the description of consumer groups and is able to more fully examine the factors influencing user behavior than the UTAUT model, which focuses on the employees of the company.

Many theories and models have been developed in order to better comprehend the factors affecting the adoption-acceptance of new technologies. The UTAUT is one of the most comprehensive models of technology acceptance since it integrats TIF, TRA, TPB, SCT, IDT, MPCU, MM, composite TAM&TPB and other theories of technology acceptance in information technology research. It consists of four factors (Performance Expectancy, Effort Expectancy, Social Influence, Facilitating Conditions) and four moderating variables (Gender, Age, Experience, Voluntariness of use) that directly affect the use intention and behavior (Venkatesh et al., 2003; Kan, 2016). In the context of mobile learning, UTAUT was suggested as the best possible mode (Venkataraman and Ramasamy, 2018; Jiang, 2021). However, there is a lack of consideration of consumer habits, payment prices and other factors in different consumer centric work environments. Thus, UTAUT2 emerged as an extension of UTAUT, incorporating Hedonic Motivation (HM), Price Value (PV), and Habit (HT). Behavioral Intention (BI) is the mediating variable, while Use Behavior (B) is the dependent variable (Venkatesh et al., 2012). At present, this model has been verified in personal consumption, online learning, e-government, online education, etc., (El-Masri and Tarhini, 2017; Chaiyasoonthorn and Suksa-Ngiam, 2018; Eneizan et al., 2019; Basewe et al., 2022). Digital academic reading includes technical factors (ICT technology), reading attitudes, behavioral factors, and digital environmental factors, which roughly fit the UTAUT2 model. Therefore, this paper combines it with digital academic reading theory, replacing appropriate variables and increasing the explanatory power of the model.

### 3.2. Model construction

**Figure 2:**
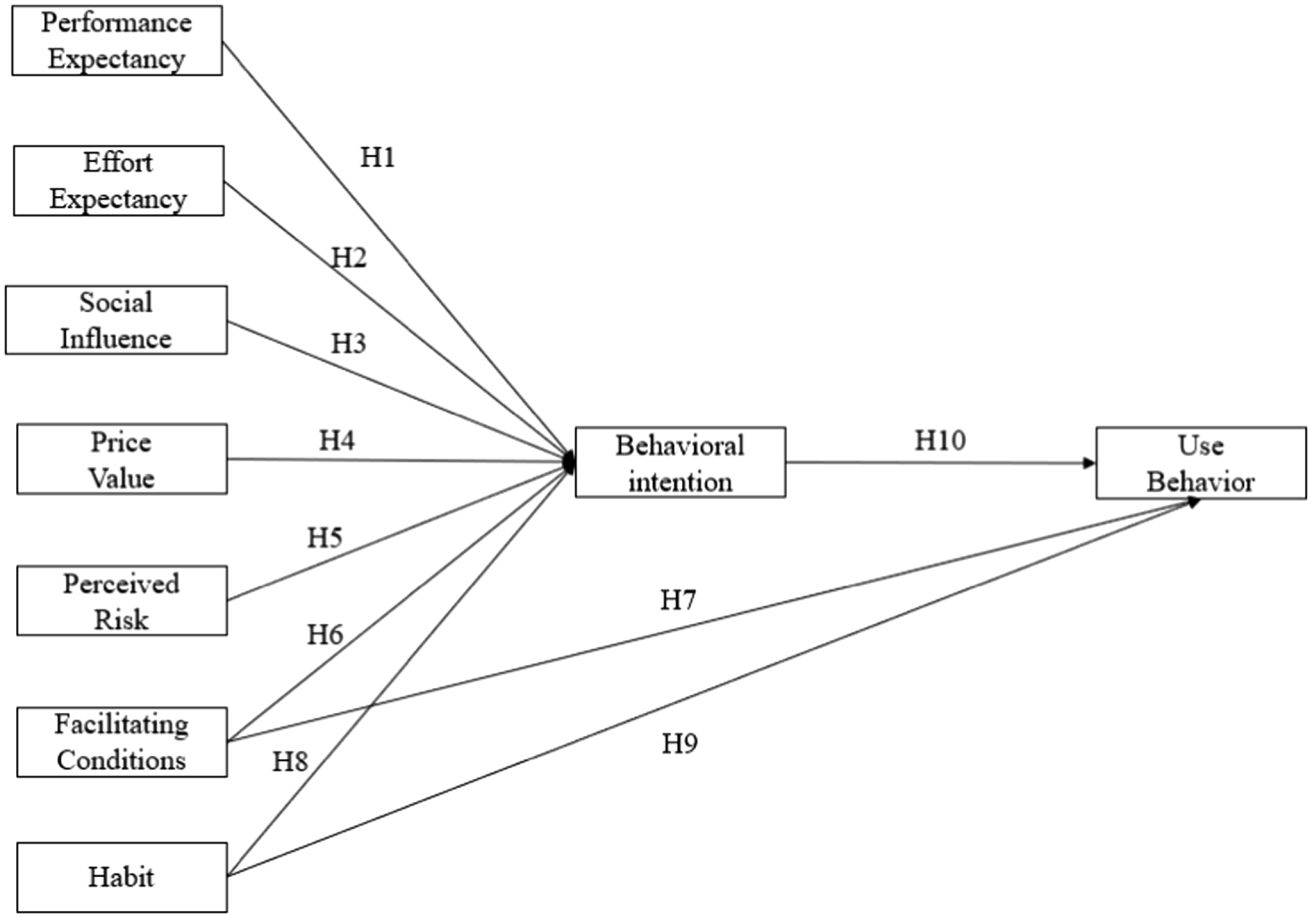
DARB model of college students.

Proposing a theoretical framework to study the mechanism influencing college students’ digital academic reading behavior is a tough undertaking to ensure that the model is comprehensive and concise, and excluding variables of little value from the research. Academic reading requires the use of reading strategies suitable for specific disciplines to read professional academic resources (McWhorter, 2013), which is a purposeful (Mayer and Alexander, 2011), critical and complex reading behavior (Sengupta, 2002). It usually requires readers to deeply understand and synthesize densely scattered texts, and requires them to concentrate on reading. Therefore, the motivation for academic reading is mainly scientific research or academic improvement, rather than hedonic motivation in leisure or general consumption, so this influencing factor should be removed. In the digital environment, readers will encounter network risks in the reading process, such as advertising interference, false information (Pang, 2019), personal distraction (Zhou, 2019), personal information disclosure, etc. Therefore, perceived risk is added to explore the impact of individual cognitive risk on use intention and behavior in digital academic reading. The original message of the UTAUT2 is that gender, age, and experience are normally set to moderate UTAUT2 relationships (Venkatesh et al., 2012). However, several studies conducted *via* UTAUT and UTAUT2 in various IS/online learning have reported to some extent contradictory findings (Chauhan and Jaiswal, 2016; Alajmi and Alotaibi, 2020; Jaradat et al., 2020). In addition, many studies in the field of education have excluded these three moderating variables for various reasons In addition, many studies in the field of education have excluded the three moderate variables for various reasons (Ahmed and Ali, 2020; Zacharis and Nikolopoulou, 2022). In china, students are or should be able to read proficiently by the time they enter university and there is a small gap between college students’ performance and reading experience. Moreover, the universities attach importance to the development of information literacy, and Chinese universities have basically carried out similar information literacy courses in the first academic year, with students of different ages and genders acquiring the same information skills. Undergraduate students’ academic reading experience mainly comes from course assignments. Therefore, their digital reading experiences are roughly the same. At the same time, this study focuses on digital academic reading behavior rather than reading performance. Therefore, the three regulatory variables of gender, age and experience are not considered. Finally, the digital academic reading information behavior (DARB) model of college students is shown in Figure 2.

### 3.3. Research hypotheses

Performance expectancy refers to the extent to which an individual believes that their performance can be enhanced by the new system or technology (Venkatesh et al., 2003). In the context of DAR (digital academic reading), It is believed that digital academic reading can help readers obtain academic information resources of better quality faster and more efficiently. Studies have shown that performance expectancy (PE) has a significant influence on the behavioral intention (BI). A growing number of scholars found that PE is a significant predictor of BI in the context of mobile learning (Wang et al., 2009; Fagan, 2019). PE was also shown to predict BI of primary school teachers’ use of technology use (Lufungulo, 2015), as well as BI of ICT instruction in a forced environment in the Philippines (Kim and Lee, 2020). PE also positively affected the BI of college students using mobile learning technologies in China, in the United States, and in Indonesia (Chao, 2019; Fagan, 2019; Sidik and Syafar, 2020), as well as mobile internet technology in India and Germany (Jayanth and Murugan, 2020). Digital academic reading falls under the category of online learning. Based on the findings of online learning, this study hypothesizes that performance expectancy (PE) positively affects college students’ behavioral intention (BI) to engage in digital academic reading:

*H*1: Performance expectancy (PE) has a significant positive effect on behavioral intention (BI).

Effort expectancy is defined as the expected ease of using the technology (Paula et al., 2021). In the context of this study, it refers to the ability of college students to quickly master and easily operate digital academic reading tools. Since then, effort expectancy (EE) has always been important influencing factor of behavioral intention (BI) and use behavior (USE) in the research of education and tourism (Baptista and Oliveira, 2015). Scholars found that EE can influence secondary school teachers’ intentions to use mobile technology, or broader technology in the classroom (Omar et al., 2019; Kim and Lee, 2020). But Botero et al. (2019) found no effect of effort expectancy on attitude and behavioral intention in a m-learning study. These inconsistent findings require more empirical studies to better explain the role of effort expectancy in digital academic reading. Therefore, the following hypothesis was formulated:

*H*2: Effort expectancy (EE) has a significant positive effect on behavioral intention (BI).

Social Influence refers to the perception that significant others believe that the technology should be used (Paula et al., 2021). Social influence played an important role in explaining behavioral intention in m-learning studies (Botero et al., 2019). Social Influence (SI) affected teachers’ Behavioral Intention (BI) to use technology (including mobile technology) in the classroom (Kim and Lee, 2020), as well as college students’ BI to use mobile learning technologies (Alasmari and Zhang, 2019; Jayanth and Murugan, 2020). During the COVID-19 Pandemic, students have invested a lot of time in digital reading. Students’ DAR (digital academic reading) intention may be influenced by other important individuals, such as friends, teachers, or family. In the present study, it was hypothesized that:

*H*3: Social influence (SI) has a significant positive effect on behavioral intention (BI).

Studies have shown that price value (PV) has a significant influence on the behavioral intention (BI). (Wong et al., 2019; Kleopatra et al., 2021). In this study, price value (PV) refers to the amount of time, energy and money that college students need to spend on digital academic reading (Venkatesh et al., 2003; Huang, 2019). Sensible or low-cost online access (as well as technical devices’ purchase) may influence students’ intention to conduct digital academic reading. Higher PV is expected to be linked to greater willingness to adopt digital academic reading. Therefore, we put forward the following hypothesis:

*H*4: Price value (PV) has a significant positive effect on behavioral intention (BI).

Perceived risk (PR) refers to online risks encountered in digital academic reading, such as personal privacy security, online advertising or pop-up interference. Many scholars have confirmed its negative effect on people’s behavioral intention (BI) to conduct digital academic reading. For example, Thomas and Baird (1985) believed that the uncertainty of information sources in digital academic reading would affect the quality of information obtained. Deng et al. (2015) also proved that advertisements and pop-ups in the process of digital academic reading affected the quality of information. According to the previous researches, the hypothesis is as follows:

*H*5: Perceived risk (PR) has a significant negative effect on behavioral intention (BI).

Facilitating conditions were defined as the technical or organizational support expected while using the technology. Facilitating Conditions (FC) can significantly predict behavioral intention (BI) and positively influence students’ digital reading behavior (Taiminen and Karjaluoto, 2017; Dwivedi et al., 2019; Jayanth and Murugan, 2020). Kim et al. (2005) believed that digital academic reading can make better use of fragmented time. FC also predicted educators’ and students’ BI to adopt and use mobile internet (Wong et al., 2019). For this study, it was hypothesized that FC affect students’ BI to conduct digital academic reading. Technical (e.g., restricted internet access and limited technology infrastructure) and organizational issues (e.g., lack of support personnel) may possibly inhibit students’ intentions and usage in academic reading. Students’ intention to engage in digital academic reading is stronger when they perceive that the school has adequate support and the right environment for Internet implementation. The related research hypotheses are as follows:

*H*6: Facilitating conditions (FC) has a significant positive effect on behavioral intention (BI).

*H*7: Facilitating conditions (FC) has a significant positive effect on use behavior (B).

Habit (BH) refers to the transformation of college students’ information reading habit from traditional paper academic reading to academic reading under the network environment. The positive relationship between BH and students’ intentions to use technology was confirmed by Omar et al. (2019). BH had positive effect on teachers’ behavioral intention to use actual technology (Kim and Lee, 2020), and it influenced educators’ and students’ BI to adopt and use mobile internet (Wong et al., 2019). Scholars have also found that students’ academic reading habits have changed due to digital reading in the online environment (Huang, 2019). This study hypothesized that habit has a positive effect on students’ (Behavioral Intention) BI and Use Behavior (B) for digital academic reading. Students’ digital academic reading habits may contribute to their increasing receptive and academic competence. The related hypotheses are as follows:

*H*8: Habit (BH) has a significant positive effect on behavioral intention (BI).

*H*9: Habit (BH) has a significant positive effect on use behavior (B).

Previous researches have proved that there is a positive relationship between behavioral intention and use behavior (Jayanth and Murugan, 2020; Kim and Lee, 2020). Most students are willing to use digital academic reading to obtain information if possible (Venkatesh et al., 2003; Nie, 2019). This study hypothesized that BI positively affects students’ actual use of digital academic reading. The higher students’ intentions possibly relate to higher usage for academic reading purposes. Therefore, we put forward the hypothesis:

*H*10: Behavioral intention (BI) has a significant positive effect on use behavior (B).

## 4. Methodology

### 4.1. Participants

In this study, in order to ensure the balance of sampling (age, specialty, etc.), a multi-stage sampling technique was used to collect representative samples from universities in Nanjing. Initially, two schools were randomly selected from five university towns divided by Nanjing Municipal Education Bureau. Then, up to 10 colleges (five science colleges and five humanities colleges) were randomly selected from each college. From each university, one major was randomly selected to participate in the current study.

Two hundred thirty-nine college students participated in this study, of which 189 students’ data is deemed valid after eliminating those with obvious filling errors (e.g., identical answers from beginning to end, or too many missing values in the questionnaire). The classification of college students is as follows: The classification of college students is as follows: (1) 83 undergraduate students in the humanities, (2) 106 undergraduate students in the sciences and the ratio of arts and sciences disciplines is close to 1:1. Most students learn the basic professional knowledge of their major by reading academic articles online.

### 4.2. Study materials

The questionnaire in this study is a closed questionnaire, modified from the pre-survey questionnaire reliability and validity. The questionnaire is divided into two parts. The first part collects the basic information of students: gender, age, grade, habits and overall situation of digital academic reading. After the completion of the scale design, a pre-survey was conducted among 30 college students who often conduct digital academic reading, and the official questionnaire was formed by discussing, modifying and adjusting problem areas. And it was adopted to measure the respondents’ behavioral intention of digital academic reading and its influencing factors (Xie, 2012; Li et al., 2014). The questionnaire uses a Likert 5-point scale with values ranging from “strongly disagree” to “strongly agree” (Supplementary Appendix 1).

### 4.3. Data collection

In addition, demographic data were collected by grade and discipline. The data was collected through a third-party online survey software “questionnaire star”,^1^ which was not connected to any of the college systems that collected the research sample. In order to protect the confidentiality and anonymity of the respondents, the questionnaire data did not collect the names, email addresses or phone numbers of the respondents. In addition, demographic data are collected by grade and subject.

Prior to data collection, the School of Management of Nanjing University of Posts and Telecommunications issued an official license for the project, so as to issue large-scale online questionnaires. Before filling in the questionnaire, participants were informed of the survey intention and explained the meaning and content of digital academic reading to ensure the respondents’ understanding of the questionnaire and the authenticity of data reflecting users’ digital academic reading behavior. The appraisers of the project should collect the questionnaire from April 24 to May 1, 2022 and complete the screening checks in time. It should also be mentioned that students were told that if they did not want to fill out the questionnaire, they could ask to exclude themselves. Finally, the students were given the contact details of the researcher in case they wanted more clarification.

## 5. Results

### 5.1. Demographic information

A survey of the digital academic reading tools, frequency, duration, location and content used by college students provides insight into the academic reading habits of college students in a digital environment.

In terms of reading carriers, the most used carriers are computers, followed by mobile phones, and the least are e-book readers. One hundred nine of them use two or more reading carriers, accounting for about 57.7% of the total. In terms of reading frequency, the number of students who read “frequently” is the lowest, only 21(11.1%), and 50% of them were seniors and postgraduates. The number of students with “occasional” frequency is the largest, totaling 100(52.9%), and “hardly” frequency accounts for 20.1%. In terms of the length of single reading, the majority of the respondents read for less than 1 hour, accounting for 79.9% of all the respondents, while 5.3% of them read more than 2 hours. In terms of reading places, the largest number of students choose dormitories and libraries for academic reading, accounting for 85.7%, and only two students choose cafes. In terms of reading channels, university students use search engines such as Baidu and Google the most (27.2%), followed by library databases (24.6%), professional websites (21.2%), academic platforms (13.9%), and social networks the least (13.1%). The highest percentage of new undergraduate students used search engines (30.4%) In terms of reading content, students read mainly Chinese literature (89.9%), with only a small proportion of foreign literature.

### 5.2. Reliability and validity analysis

The reliability of the scale is determined by the internal consistency coefficient (Cronbach’s α), average variance extracted (AVE) and composite reliability (CR). As shown in Table 2, Cronbach’s α of Performance Expectancy, Social Influence, Price Value, Perceived Risk, Facilitating Conditions, Habit, Behavioral Intention, Use Behavior are greater than 0.7, the AVE of the factor load value of each measurement item is greater than 0.5, and the CR of each combination reliability is greater than 0.7, indicating that each measurement item of the questionnaire has very good reliability. Validity testing includes content validity and structural validity. The design of this study’s scale was refined and modified through literature research, expert interviews and pre-survey of items. The process is rigorous and has good content validity. The KMO is 0.934, greater than 0.8, the Bartlett’s sphericity value is 344.692 (DF = 196), and the statistical significance (P) is less than 0.001, indicating that the research data has high correlation and is suitable for factor analysis. In confirmatory factor analysis (CFA), the factor load value of each item is greater than 0.6, indicating that the validity of the measurement model is good.

**Table 2:** Reliability and validity analysis results of questionnaire.

Dimension	Item	Factor loadings	SMC	CR	AVE	Cronbach’s *α*
Performance expectancy	PE1	0.899	0.808	0.8925	0.7348	0.892
	PE2	0.845	0.714			
	PE3	0.826	0.683			
	EE2	0.847	0.718	0.8789	0.7828	0.872
	EE3	0.921	0.848			
Social influence	SI1	0.805	0.648	0.7267	0.5718	0.723
	SI3	0.704	0.496			
Price value	PV1	0.881	0.776	0.8226	0.6127	0.812
	PV2	0.832	0.692			
	PV3	0.608	0.370			
Perceived risk	PR1	0.921	0.848	0.8679	0.6901	0.863
	PR2	0.877	0.768			
	PR3	0.673	0.453			
Facilitating conditions	FC1	0.858	0.735	0.8479	0.6510	0.851
	FC2	0.740	0.547			
	FC3	0.818	0.67			
Habit	BH1	0.865	0.749	0.8481	0.6519	0.850
	BH2	0.831	0.691			
	BH3	0.719	0.517			
Behavioral intention	BI1	0.882	0.778	0.8896	0.8012	0.892
	BI2	0.908	0.824			
Use behavior	B1	0.657	0.431	0.7937	0.6652	0.773
	B3	0.948	0.899			

### 5.3. Structural model

Observation variables are continuous and follow normal distribution, and potential variables have significant correlation, so SEM method can be used for model fitting (Supplementary Appendix 2). Amos26.0 is used to study the overall fitting evaluation and hypothesis test of the model. It can be seen from Table 3 that the model fitness meets the standard, indicating that the data collected and the model constructed match well, the proposed path assumption relationship is consistent with the actual situation, and the model coefficient results are accurate and effective.

**Table 3:** Model fitness parameters.

Fitting coefficient	Evaluation criterion		Actual value	Fitting situation
	Good	Acceptable		
X2/DF	<3	3.0–5.0	1.759	Good
GFI	>0.9	0.7–0.9	0.865	Acceptable
AGFI	>0.9	0.7–0.9	0.809	Acceptable
CFI	>0.9	0.7–0.9	0.958	Good
RMR	Close to 0	<0.5	0.027	Acceptable
TLI	>0.9	0.7–0.9	0.946	Good
RMSEA	<0.08	0.08–0.1	0.064	Good

The degree of interpretation of the whole model and the significance of relevant assumptions are evaluated by path coefficient, C.R. value and *p* value, as shown in Table 4. The absolute value of the critical ratio C.R. of 7 paths is greater than 1.96, and the significance probability value p is less than 0.05; these hypotheses are accepted. The absolute value of the critical ratio C.R. of 3 paths is less than 1.96, and the significance probability value *p* is greater than 0.05; these hypotheses are rejected. The verification results in Table 4 show that some hypotheses proposed in this paper have passed the test.

**Table 4:** Analysis of model results.

Hypothesis	Path	Unstandardized regressive coefficient	S.E.	C.R.	*P*	Standardized regressive coefficient	Result
H1	BI ← PE	0.049	0.095	0.517	0.605	0.053	Rejected
H2	BI ← EE	0.338	0.163	2.074	0.038	0.348	Accepted
H3	BI ← SI	−0.330	0.166	−1.98	0.048	−0.307	Accepted
H4	BI ← PV	0.329	0.124	2.644	0.008	0.334	Accepted
H5	BI ← PR	−0.472	0.241	−1.962	0.050	−0.469	Accepted
H6	BI ← FC	0.184	0.141	1.302	0.193	0.180	Rejected
H7	B ← FC	0.372	0.182	2.046	0.041	0.369	Accepted
H8	BI ← BH	0.943	0.308	3.067	0.002	0.905	Accepted
H9	B ← BH	0.146	0.138	1.06	0.289	0.142	Rejected
H10	B ← BI	0.471	0.116	4.054	***	0.448	Accepted

***corresponds to 0.1% significant level, *p* < 0.001.

**Figure 3:**
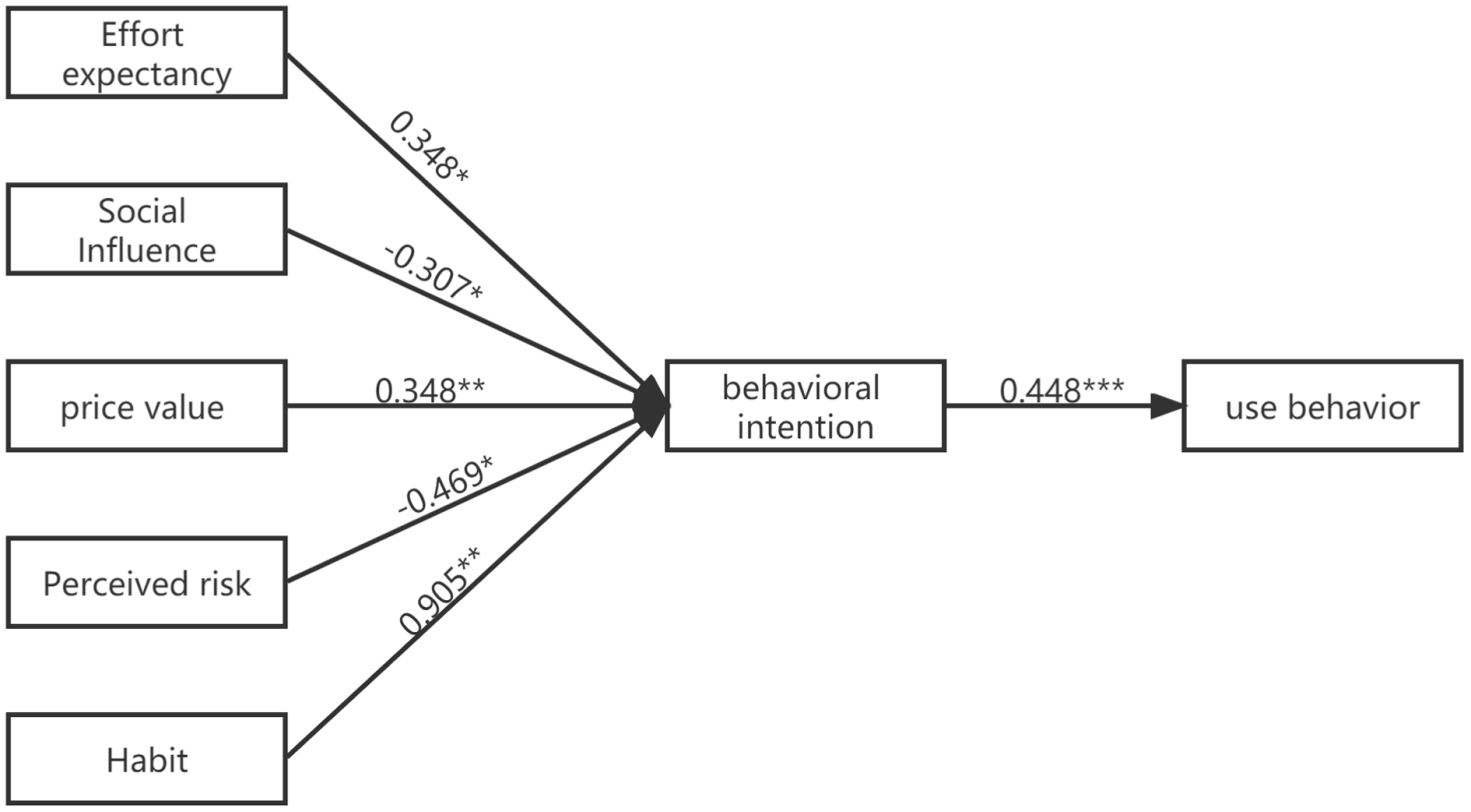
Digital academic reading behavior model of college students.

In terms of model modification, the index variable whose factor load was less than 0.6 was deleted, and some index variables were adjusted according to the MI value. The index variable corresponding to the error variable with a larger MI value was deleted, and the correlation between the other error variables was added to obtain the new model. After empirical research and modification, the final digital academic reading behavior model (DARB) of college students is shown in Figure 3.

## 6. Discussion

The data suggests that undergraduates have recognized the importance of academic reading. However, most students do not place much emphasis on academic reading, and the demand for it is not high. Some senior students have gradually increased the reading frequency of academic literature because of project submissions and thesis writing. What’s more, digital academic reading requires a lot of energy, and it remains difficult for college students to read professionally for a long time. And the majority of students choose to do their academic reading on campus, which should be related to the need to access the campus network for digital academic reading. Finally, after the introduction of information retrieval and analysis courses in many colleges in the second year of university, college students learn that they can obtain digital academic resources from the database resources of digital library and their academic reading ability is also growing with the grade.

Based on the UTAUT2 model, this study adjusted the explanatory variables according to the characteristics of digital academic reading, removed hedonic motivation, gender, age and experience variables, added perceived risk (PR), and constructed the college students’ digital academic reading information behavior model (DARB). Amos 26.0 software is used to analyze the survey data, and it is found that effort expectancy (EE), social influence (SI), price value (PV), perceived risk (PR) and habit (BH) have a significant effect on behavioral intention (BI), and behavioral intention (BI) has a significant effect on use behavior (B). However, performance expectancy (PE) and facilitating conditions (FC) have no significant influence on behavioral intention (BI) and use behavior (B). The results of this analysis contradict with previous studies (Alsaaid and Abd Razak, 2020; Farshad, 2022). When students believe that the educational process is beneficial to them, their willingness to use it will increase significantly. According to the survey results, the main concern of undergraduate students is to improve their academic performance. They regard digital academic reading as an activity that can help them do this. However, in this study, the students’ responses showed that the expectation performance did not affect their behavioral intention of digital academic reading. The main reason for this may be that students were not aware of the benefits of digital academic reading. At the same time, some students were bored with the frequent digital academic reading behavior during covid-19 epidemic. Even though they thought that the reading process could benefit them, they were reluctant to initiate the reading process. For the same reason, students’ willingness to read did not improve even when they were provided with easy access to digital academic reading.

Effort expectancy has a significant positive effect on behavioral intention. This is consistent with the hypothesis in the original UTAUT2 model (Venkatesh et al., 2012; Omar et al., 2019; Kim and Lee, 2020). It is show that the convenience, system stability and difficulty of digital academic reading affect users’ behavioral intention. With the construction and development of the digital academic reading database platform, the operation of the system is becoming more and more convenient, stable and durable, reducing the efforts of college students to use the digital reading system, and the intention to read digital academic work will increase; On the contrary, if the system is complex and cumbersome, the intention will decline.

Habit is another most important factor that influences behavioral intentions and use behavior. Habit reflects users’ dependence on new information skills. Academic reading ability is a reflection of college students’ information literacy. Most students must be exposed to digital academic reading and have ability to obtain and use digital information, so they have a strong dependence on it.

Social influence has a significant negative effect on behavioral intention. This is inconsistent with the positive impact of previous studies (Gil-Flores et al., 2012; Hahnel et al., 2016; Lim and Jung, 2019). But some studies also show that social influence has no effect on behavioral intention (Kleopatra et al., 2021). Social influence reflects the influence of individual social environment on digital academic reading. Based on the questionnaire content and the results of face-to-face interviews with some college students, it is found that modern college students pursue independence and freedom, hate preaching, and they are unique, especially affected by the COVID-19 for a long time, making digital academic reading a regular learning content. The compulsion of learning tasks and the monotony of the reading platform (for example, students point out that they often use the pdf format documents published by teachers on the online platform stipulated by the school) reduce students’ interest in digital academic reading and hold a negative attitude. Therefore, when teachers or classmates around encourage students to carry out academic reading, they will arouse their aversion to digital academic reading.

Price value also has a significant positive impact on behavioral intention. This is consistent with previous studies (Wong et al., 2019; Kleopatra et al., 2021). With the accelerated construction of digital libraries, most college libraries have sufficient digital academic resources, and provide students with perfect reading equipment and convenient access to the Internet (Fang and Jia, 2019). The cost of students’ digital academic reading is greatly reduced. In addition, colleges tend to provide a large number of courses related to information resource retrieval, academic report writing, and academic paper reading. Therefore, students have gradually developed their interest in digital academic reading, which has also greatly improved the utilization of digital academic resources.

Perceived risk also has a significant negative impact on behavioral intention. Perceived risk reflects the risk that college students think digital academic reading may pose to them. Digital academic reading may pose risks to users in terms information quality, such as Pop up Window, personal information disclosure and so on, which have a great effect on college students (Mizrachi, 2015; Clair-Thompson et al., 2017).

The hypothesis of the influence of performance expectation on behavioral intention is not valid, unlike the results of most previous studies (Gil-Flores et al., 2012; Venkatesh et al., 2012; Hahnel et al., 2016; Lim and Jung, 2019), and is very worth discussing. Based on the content of the questionnaire and the results of face-to-face interviews with some college students, it is found that the digital academic reading of undergraduate students mainly occurs in their senior year, spent entirely on the graduation design and graduation thesis, and has a strong purpose. However, the importance of digital academic reading is ignored during the freshman to junior year, resulting in a non-significant impact of performance expectancy on behavioral intention. According to the analysis of questionnaires and interviews. Firstly, 57.6% of college students’ single digital academic reading time is more than half an hour, which is time-consuming. Academic reading is deep reading and requires a long period of time, and so fragmented time cannot be used reasonably and effectively. Secondly, 85.7% of students choose to do digital academic reading in libraries and dormitories. Compared with other public places, the learning atmosphere and environment are better and easier for them to concentrate. In addition, influenced by pandemic prevention and control, college students can only choose to study on campus, and the space convenience is also limited. Therefore, the influence of convenience conditions on behavioral intention and use behavior is not significant.

## 7. Conclusion and limitations

The main limitations of the study include the convenience sample and the use of a self-reporting scale to assess student’ behavioral intention and use behavior. Due to the limitation of covid-19 epidemic prevention measures, the samples were mainly from college students in Nanjing. The sample lacks diversity and may suffer from selection bias (Kleopatra et al., 2021). Secondly, this study is cross-sectional in nature, so it does not model the change of students’ digital academic reading intention and behavior over time. To validate these patterns, more studies could be carried out with representative samples. For example, more geographical samples should be included. In addition, the sample should involve a more academically focused sample of postgraduate students, and explore whether the model can explain the amount of digital academic reading behavior of graduate students.

Despite the aforementioned limitations, this study produced promising findings. Overall, the findings supported the use of the modified UTAUT2 model as an appropriate framework for Digital Academic Reading Behavior. This offers new perspectives on the role of perceived risk (PR) on students’ behavior of digital academic reading in the context of Covid-19 popular, and calls for more empirical evidence on the role of social influence in the UTAUT model.

High level of Effort expectancy and low price value can effectively improve the reading intention of college students. Therefore, it is suggested that colleges should establish a training system for digital academic reading literacy and information literacy as soon as possible, cultivate their ability to acquire and use information and develop their keen information insight, strengthen professional information awareness, improve professional in-depth learning knowledge and enhance academic literacy, improve students’ effort to find digital academic resources accurately and effectively on the Internet, and reduce the cost of obtaining digital academic information. In addition, the operation system, digital academic reading resources and reading environment of university digital library system should be optimized to raise the expectation of students’ efforts to read digitally on the platform.

Finally, it is necessary to strengthen the construction of library digital resources, focus on the source supervision and quality control of digital academic resources, reasonably integrate digital academic resources to improve the availability of digital academic resources, provide an open, safe and stable working environment, and reduce students’ investment and expenditure in digital academic reading. It is also suggested that the security protection of personal information on the internet should be strengthened to prevent the disclosure of personal information during the use of resources and to reduce the perceived risk of users.

## Data availability statement

The original contributions presented in the study are included in the article/Supplementary material, further inquiries can be directed to the corresponding author.

## Ethics statement

In accordance with local legislation and institutional requirements, the questionnaire can only be carried out after fully explaining the study to the participants and obtaining their agreement to participate. Neither the survey process nor the results will involve the disclosure of participants’ personally identifiable information.

## Author contributions

LC and JL: conceptualization and supervision. JL: methodology and writing—review and editing. YW and YF: software and writing—original draft preparation. LC: validation and project administration. YF: investigation. XZ: visualization All authors have read and agreed to the published version of the manuscript.

## Conflict of interest

The authors declare that the research was conducted in the absence of any commercial or financial relationships that could be construed as a potential conflict of interest.

## Publisher’s note

All claims expressed in this article are solely those of the authors and do not necessarily represent those of their affiliated organizations, or those of the publisher, the editors and the reviewers. Any product that may be evaluated in this article, or claim that may be made by its manufacturer, is not guaranteed or endorsed by the publisher.

## Footnotes

^1^ www.wjx.cn
