# Supplementary material for: Study on factors influencing college students’ digital academic reading behavior
Source: Front Psychol. 2023 Jan 12;13:1007247. doi: 10.3389/fpsyg.2022.1007247 (PMC9877342; doi:10.3389/fpsyg.2022.1007247)
Supplement: Supplementary file 1 [file Data_Sheet_1.PDF]

Appendix 1

A questionnaire survey on college students' digital academic reading behavior

Dear students:

I am a student of the School of Management of Nanjing University of Posts and Telecommunications. If you want to know the digital academic reading habits of college students, please fill in the following questionnaire for about three minutes. Thank you very much for your support. I wish you success in your studies and work! This survey is conducted anonymously and will not collect any of your private information and will not be used for any commercial purposes.

In this questionnaire, digital academic reading refers to reading professional books, papers, abstracts of international conference proceedings, and content on academic forums through digital carriers (mobile phones, tablets, computers), including reading behavior to solve professional problems and obtain information from knowledge communities such as Zhihu and CSDN.

I. This part of the questionnaire is a basic information survey.

1.What's your gender?

A. Male

B. Female

2.What's your grade?

A. Freshman

B. Sophomore

C. Junior

D. Senior

E. Grade 1 master

F. Grade 2 master

G. Grade 3 master

3. What is your subject category?

A. Natural science (science, engineering, agriculture, medicine)

B. Humanities

4. What electronic equipment do you usually use when reading academic articles, papers or works? (Multiple choices)

A. Mobile phone

B. Flat plate

C. Computer

D. E-reader

5. How often do you do digital academic reading?

A. Frequently (5 or more times per week)

B. Often (3-4 times a week)

C. Occasionally (1-2 times a week)

D. Almost not

6. What is the average length of a single digital academic reading?

A. Within 30 minutes

B. 30 minutes to 1 hour

C. 1 hour to 2 hours

D. More than 2 hours

7. Where do you usually conduct digital academic reading?

A. Dormitory

B. Library

C. Classroom

D. Coffee shops and other public places

8. What are your main ways to conduct digital academic reading? (Multiple choices)

A. Library database

B. Professional subject websites

C. Baidu, Google and other search engines

D. Social networking

E. Academic Forum

9. What are the main contents of your digital academic reading? (Multiple choices)

A. Leading edge of discipline

B. Research Methods

C. Relevant background knowledge

D. Tool Technology

E. Basic professional knowledge

F. Hot knowledge

10. What is the main language of academic articles, papers or works that you use mobile phones, computers and other devices to retrieve?

A. Chinese

B. English

C. Other

II. This section is about your statements in the process of digital academic reading. Please choose the most appropriate option according to your personal views or feelings. Among them, 1 represents disagree very much, 2 represents disagree relatively, 3 represents average, 4 represents agree relatively, and 5 represents agree very much.

11.Through the following questions, we would like to know your views on the performance expectations of digital academic reading.

Number	Subject	1	2	3	4	5
1	Digital academic reading enables me to get information faster.					
2	Digital academic reading enables me to obtain more and broader information.					
3	Digital academic reading can improve my learning efficiency.					

12.Through the following questions, we would like to know your views on the expectations of digital academic reading efforts.

Number	Subject	1	2	3	4	5

1	The tools used for digital academic reading are more mobile and convenient.					
2	The system used for digital academic reading is stable and easy to operate.					
3	Through the digital academic reading system, I can easily obtain the information I want.					

13.Through the following questions, we want to understand the impact of social environment on your digital academic reading.

Number	Subject	1	2	3	4	5
1	If all the people around me conduct digital academic reading, I will do the same.					
2	Digital academic reading enables me to better integrate with the people around me.					
3	Digital academic reading makes my opinion more authoritative.					

14.Through the following questions, we would like to know your views on digital academic reading in terms of convenience.

Number	Subject	1	2	3	4	5
1	Digital academic reading can make better use of fragmented time.					
2	Now I can do digital academic reading on various occasions.					
3	Digital academic reading equipment is easy to find.					

15.Through the following questions, we would like to know your views on the cost of digital academic reading.

Number	Subject	1	2	3	4	5
1	Academic reading with numbers saves time.					
2	Academic reading with numbers saves energy.					
3	Less money spent on digital academic reading.					

16.Through the following questions, we would like to know your habits of digital academic reading in the network

environment.

Number	Subject	1	2	3	4	5
1	I am more accustomed to digital academic reading.					
2	In today's network environment, my academic reading habits have changed due to digital reading.					
3	Digital academic reading has become my habit.					

17.Through the following questions, we would like to know your perception of the possible risks of digital academic reading.

Number	Subject	1	2	3	4	5
1	The unknown source of information in digital academic reading will affect the quality of information I get.					
2	Advertisements and pop ups in the process of digital academic reading will affect the quality of information I get.					
3	Personal information leakage caused by digital academic reading makes me uneasy.					

18.We would like to know your willingness to use digital academic reading through the following questions.

Number	Subject	1	2	3	4	5
1	I am willing to learn to use numbers for academic reading.					
2	If feasible, I would like to use digital academic reading to obtain information in the future.					
3	I am willing to recommend digital academic reading to people around me.					

19.Through the following questions, we want to know your digital academic reading use behavior.

Number	Subject	1	2	3	4	5
1	I often do digital academic reading.					
2	I often recommend digital academic reading to others.					

3	I will continue to do digital academic reading in the future.	

Thank you for your participation!
