# Supplementary material for: Study on factors influencing college students’ digital academic reading behavior
Source: Front Psychol. 2023 Jan 12;13:1007247. doi: 10.3389/fpsyg.2022.1007247 (PMC9877342; doi:10.3389/fpsyg.2022.1007247)
Supplement: Supplementary file 2 [file Data_Sheet_2.PDF]

Appendix 2

Table Correlation coefficient between potential variables

	Estimate	C.R.	P
PE <--> EE	0.427	7.535	***
PE <--> SI	0.297	5.748	***
PE <--> PV	0.336	6.594	***
PE <--> PR	0.349	6.339	***
PE <--> FC	0.370	6.828	***
PE <--> H	0.341	6.650	***
EE <--> SI	0.313	6.276	***
EE <--> PV	0.397	7.732	***
EE <--> PR	0.395	7.135	***
EE <--> FC	0.410	7.619	***
EE <--> H	0.349	7.068	***
SI <--> PV	0.343	6.908	***
SI <--> PR	0.335	6.390	***
SI <--> FC	0.304	6.072	***
SI <--> H	0.370	7.107	***
PV <--> PR	0.356	6.916	***
PV <--> FC	0.386	7.443	***
PV <--> H	0.424	8.083	***
PR <--> FC	0.401	7.130	***
PR <--> H	0.362	7.012	***
FC <--> H	0.356	7.106	***

Test of normal distribution of the questionnaire

Influencing Factors	Average Value	Standard Deviation	Skewness	Kurtosis
PE1	3.87	0.843	-1.036	2.053
PE2	3.95	0.855	-0.930	1.561
PE3	3.80	0.798	-0.648	1.218
EE1	3.99	0.796	-0.887	1.519
EE2	3.92	0.746	-0.716	1.609
EE3	3.90	0.786	-0.692	1.140
SI1	3.74	0.815	-0.611	0.936
SI2	3.53	0.789	-0.261	0.307
SI3	3.75	0.790	-0.443	0.624
FC1	3.80	0.807	-0.601	0.748
FC2	3.72	0.870	-0.403	-0.190
FC3	3.81	0.807	-0.508	0.607

PV1	3.76	0.759	-0.678	1.604
PV2	3.66	0.780	-0.414	0.596
PV3	3.71	0.809	-0.884	1.236
BH1	3.78	0.781	-0.807	1.587
BH2	3.79	0.789	-0.733	1.429
BH3	3.57	0.846	-0.583	0.621
PR1	3.87	0.811	-0.901	1.907
PR2	4.02	0.860	-0.944	1.554
PR3	3.69	0.870	-0.389	0.249
BI1	3.98	0.729	-1.050	3.199
BI2	3.94	0.776	-1.131	2.980
BI3	3.82	0.771	-0.732	1.651
B1	3.60	0.796	-0.247	-0.007
B2	3.34	0.882	-0.110	-0.005
B3	3.85	0.750	-0.666	1.403
